# A Culturally Tailored mHealth Intervention (MobileMen App) to Promote Physical Activity in African American Men: Protocol for a Comparative Effectiveness Trial

**DOI:** 10.2196/67809

**Published:** 2025-07-04

**Authors:** Kayla Nuss, Amanda Brice, Callie Hebert, Phillip Nauta, April J Stull, Damon L Swift, Derek M Griffith, David B Buller, Robert L Newton Jr

**Affiliations:** 1 Klein Buendel Lakewood, CO United States; 2 Population and Public Health Sciences Pennington Biomedical Research Center Baton Rouge, LA United States; 3 Department of Human Sciences and Design Robbins College of Health and Human Sciences Baylor University Waco, TX United States; 4 Department of Kinesiology School of Education and Human Development University of Virginia Charlottesville, VA United States; 5 Department of Medical Ethics and Health Policy Perelman School of Medicine University of Pennslyvania Philadelphia, PA United States

**Keywords:** physical activity, motivation, mHealth, African American men, mobile app, digital intervention

## Abstract

**Background:**

African American men are at a higher risk for serious health conditions such as cardiovascular disease, diabetes, and stroke compared to non-Hispanic White men. Physical activity (PA) is a modifiable health behavior that has been shown to decrease chronic disease risk; yet, PA engagement is alarmingly low in African American men. Interventions to improve PA engagement are effective in a number of populations; however, very few have been tailored to the unique needs of African American men. Even fewer have leveraged mobile health apps, despite African American men’s interest in and willingness to use such technologies for health improvement.

**Objective:**

This comparative effectiveness trial aims to evaluate MobileMen, a PA promotion app tailored to the needs and preferences of African American men. This trial will compare the MobileMen app to a commercially available PA promotion app with similar features but lacks culturally tailored components.

**Methods:**

We will recruit a sample of “low active” (accumulating <7500 steps per day) African American men (n=100) aged >30 years from Baton Rouge, Louisiana, and the surrounding communities. All participants are given a Fitbit Charge 6 wearable activity tracker to assess daily PA and steps and are randomized to either the MobileMen intervention app or the comparator app, which is a commercially available PA tracking app called Stridekick. The Stridekick app has features similar to those in the MobileMen app but was not intentionally designed for African American men. The intervention period is 6 months during which participants will interact with their assigned mobile app. MobileMen includes features such as digital badges earned for PA; tangible prizes like exercise equipment; challenges among participants; goal setting; nutrition; PA; and behavior change educational information in text, audio, and video formats. Participants will complete assessments at baseline and at 6 months post randomization. Assessments include objective measurements of daily steps and minutes of moderate to vigorous PA, quality of life, dietary measures, self-efficacy for fruit and vegetable consumption and PA, and autonomous motivation for PA.

**Results:**

This trial is in the start-up phase. The MobileMen app development and usability testing was completed in August 2024. Participant recruitment efforts began in October 2024. The trial and associated data analyses and interpretation are planned to be completed by fall 2025.

**Conclusions:**

Mobile apps are a widely accessible means to disseminate culturally tailored PA promotion interventions to various populations, including African American men. MobileMen has the potential to impact PA engagement in African American men, which would dramatically improve the overall health and chronic disease risk in this underrepresented group.

**Trial Registration:**

ClinicalTrials.gov NCT05621044; https://clinicaltrials.gov/study/NCT05621044

**International Registered Report Identifier (IRRID):**

PRR1-10.2196/67809

## Introduction

African American men experience disproportionately high rates of obesity, cardiovascular disease, hypertension, stroke, diabetes, and prostate cancer compared to their non-Hispanic White counterparts [1-4]. Increased levels of physical activity (PA) and fitness have been associated with the reduced risk of these chronic conditions [5-11]. However, there is a lack of regular PA in the African American community, and African American men’s rates of PA are well below national guidelines [12-15].

Comparatively, research consistently shows that African American men engage in lower levels of PA than their non-Hispanic White counterparts. Although 48% of non-Hispanic White adults meet PA guidelines of 150 minutes of moderate to vigorous activity per week, only 39% of Black adults meet that guideline [16]. This disparity is due to a complex interplay of social, environmental, and cultural factors [13,14,17,18]. Black men are more likely to live in neighborhoods with limited access to safe recreational spaces and face greater exposure to crime, which discourages outdoor activity [19]. Socioeconomic constraints such as demanding work schedules and caregiving responsibilities further limit their leisure time for PA [20]. Cultural norms around family roles, along with a lack of culturally relevant health messaging, can reduce motivation or the perceived value of PA [21,22]. Additionally, experiences of racism and discrimination—both in everyday life and within health care—contribute to chronic stress, mistrust of health institutions, and lower engagement in preventive health behaviors, including PA [23]. These structural and psychosocial barriers collectively create significant obstacles to PA for Black men. Thus, novel interventions are needed to support PA initiation and persistence in this population.

Mobile health (mHealth), which is the use of mobile phones in providing medical care, has been increasingly used to change and maintain health behaviors such as PA across various populations [24-26]. Smartphones create enormous potential for intervention delivery because they are compact, portable, normally “on,” readily available to individuals, affordable, user-friendly, offer advanced functionality, and allow online access on demand any day or time [27-32]. mHealth interventions have been used to successfully increase PA across several settings and health conditions, including in health care settings and among cancer survivors [33-36].

African American men appear to have a positive attitude toward mHealth interventions, and approximately 44% of African American men who received 5 months of PA training were willing to receive an mHealth maintenance intervention [32]. This was especially encouraging because the sample had a mean age of 53 years, and mobile app usage and acceptability is typically lower in men 50 years of age and older [32]. African American men also demonstrated a willingness to engage in mHealth interventions specifically targeting chronic disease risk reduction [32]. Moreover, evidence suggests that mHealth interventions are effective with African American men. A recent study of a comprehensive health app for African Americans indicated that users improved their minutes of moderate PA after 10 weeks of use [37]. Other studies have utilized text messaging to influence health behaviors in African Americans, also resulting in increased PA [38,39]. However, the samples in these studies were mostly females, and none of the outcome data were analyzed by sex, further elucidating the need for the development and testing of mHealth interventions specific to African American men [37-39].

To address the gap in mHealth apps developed specifically for African American men, we developed, to the best of our knowledge, a first of its kind smartphone app, MobileMen, to support PA engagement in this population. The process for developing this app and the further rationale for the selected components are described elsewhere [40]. MobileMen is grounded in 2 behavior change theories: social cognitive theory (SCT) and self-determination theory (SDT). SCT states that the environment and a person’s behavior interact to shape behavior and that self-efficacy is the key component to behavior change [41]. SCT was the foundation of the PA adoption content focusing on self-monitoring, goal setting, and social support in the MobileMen app. We chose SCT because SCT-grounded interventions have been effective at increasing and maintaining PA in African Americans [42-47]. In addition, SDT posits that satisfying 3 fundamental needs contributes to behavior change: perceived competence, a feeling of relatedness (feeling connected to others), and autonomy (feeling that change is a choice and not coerced) [48-52]. When an activity fulfills these 3 needs, people can become more autonomously motivated, resulting in more PA initiation and persistence [53]. In African American men, specifically, intrinsic motivation associates PA engagement with personal values and overall health goals, increasing the likelihood that they will persist with the behavior [54]. In the MobileMen app, we included features such as reminders and goal setting to support the development of autonomy [55]. To support competence, we included activity history and feedback such as cumulative exercise statistics aggregated over weeks and months, logging of weight training workouts and health metrics, and tangible and digital rewards [55]. We also included the ability to participate in competitions with other users and a message function to support the basic need of relatedness [55].

Additionally, the MobileMen app was culturally tailored to African American men. The culturally tailored content was based on the model established by Kreuter et al [56] who describe 5 strategies to culturally tailor an intervention: (1) peripheral, (2) evidential, (3) linguistic, (4) constituent-involving, and (5) sociocultural. Peripheral strategies involve matching the materials and messaging to the observable characteristics of the target population (eg, utilization of images of African American men), analogous to face validity and like Resnicow et al’s [57] surface structure. Evidential strategies seek to put into context the health impact for a target population (eg, notifications such as “African American men have higher rates of diabetes compared to men from other ethnic groups. Engaging in regular PA can reduce your risk of developing diabetes”). Linguistic strategies involve using language to make materials more accessible to the target audience (eg, Beast Mode badge). Constituent-involving strategies are informed by experiences or input from the population of interest (eg, utilizing focus groups with African American men). Sociocultural strategies reflect the underlying beliefs, values, and norms of a group, like Resnicow et al’s [57] deep structure. Sociocultural strategies involve how cultural, social, psychological, environmental, and historical factors affect health behaviors specifically within the target population. It is concerned with the target population’s conceptualization of the need for intervention and accurate incorporation of this information into the treatment model. Commonly noted facilitators of PA include the need for peer support and camaraderie specifically from men and competition [58,59]. African American men’s gender role responsibility and commitment to family, friends, and community also play a role in the adoption and maintenance of PA. Although the men noted these factors during focus groups for the development of the app, they could not be incorporated into the prototype due to the limited scope. However, these factors largely contribute to the current app development. For example, educational content was included that explicitly acknowledges gender role expectations and provides practical strategies based on SCT (eg, cognitive restructuring, social support techniques) and SDT (eg, fostering relatedness through competition) to assist men with incorporating family and friends into PA as part of the adoption and maintenance strategies.

The purpose of this paper is to describe the study protocol for examining the effectiveness of MobileMen. This trial will test the hypothesis that the MobileMen app will increase PA in African American men in comparison with Stridekick, a popular fitness app.

## Methods

### Study Design

A comparative effectiveness trial (CET) is being conducted to compare the effectiveness of the MobileMen app at increasing PA in African American men compared to Stridekick, a similar fitness app. The participants are assigned to one of the 2 arms: intervention (MobileMen) or comparator (Stridekick). The participants will use the assigned app on their personal smartphones for the duration of the 6-month trial. This study was registered with ClinicalTrials.gov (NCT05621044).

### Ethical Considerations

This protocol was approved by the Pennington Biomedical Research Center institutional review board on July 31, 2024 (approval 2021-052). All collected data will be deidentified before the analyses. All participants will complete an informed consent form and will receive US $200 compensation at the completion of their participation.

### MobileMen App

MobileMen is a mobile app tailored to the needs of African American men who aspire to increase their PA engagement [60]. MobileMen was developed using an extensive and iterative process described elsewhere [40]. The development of the MobileMen app was completed in July 2024 and is available for use on both iOS and Android platforms. See Table 1 for features of the MobileMen app.

**Table 1:** Features of the MobileMen and Stridekick apps.

Feature	MobileMen app	Stridekick app
Syncs to Fitbit device	✔	✔
Display physical activity data	✔	✔
Message boards	✔^a^	✔
Goal setting	✔	✔
Challenges	✔	✔
Educational content (physical activity, nutrition, and behavior change articles)	✔	N/A^b^
Allows tracking of health metrics (blood pressure, blood glucose, body weight, and cholesterol)	✔	N/A
Incentives (virtual badges and tangible prizes)	✔	✔^c^
Designed with a behavior change theory framework	✔	N/A
Designed specifically for African American men	✔	N/A

^a^MobileMen is utilizing a private Facebook group for the message board feature during the comparative effectiveness trial.

^b^N/A: not applicable.

^c^Stridekick offers virtual badges but not tangible prizes.

### Comparator App

The Stridekick app is intended to increase PA in users through a variety of functions. Stridekick is a free app available on iOS and Android platforms. See Table 1 for Stridekick features.

### Participants

Eligibility criteria include self-identification as an African American male, a resting systolic blood pressure ≤159 mm Hg and a diastolic blood pressure ≤99 mm Hg (to reduce risk of exercise-induced cardiovascular events), free of significant medical problems that would impact their ability to engage in aerobic and/or resistance training, smartphone ownership, age > 30 years, and “low active” at baseline (defined as having an average daily accelerometer-assessed step count of <7500 steps) [61].

### Recruitment and Screening

Community-based efforts, including advertising at churches, health fairs, Young Men’s Christian Association, fraternities, social media posts, and advertisements on paid, owned, and earned media, are being used to recruit study participants. Potential participants undergo a telephone screening to assess initial eligibility.

### Study Visit 1

Men who meet the initial inclusion criteria are invited for an in-person or remote orientation visit with study staff. Study staff review the study protocol and facilitate an informed consent process. During this study visit, participants complete several baseline assessments, including a sociodemographic questionnaire and height, weight, pulse, and resting blood pressure measurements. We calculate BMI by using self-reported weight and height (kg/m^2^). Health-related quality of life is assessed using the 36-item short-form health survey, a widely used validated measure with strong internal consistency (Cronbach α > 0.80 across most domains) and robust evidence of construct and criterion validity and has been validated in African Americans [62,63]. To assess PA and dietary habits, participants complete the International Physical Activity Questionnaire and National Cancer Institute’s Automated Self-Administered 24-hour Dietary Assessment Tool, respectively [64,65]. Self-efficacy for fruit and vegetable consumption is measured with 7 items that measure participants’ confidence in their ability to consume fruits and vegetables in different contexts like when away from home or in a rush [66]. Self-efficacy for PA is measured using the Self-Efficacy for Exercise Scale [67]. SDT-based motivation for PA is measured using the Behavioral Regulations in Exercise Questionnaire v.3 [68,69]. This 24-item questionnaire assesses the 6 SDT subtypes of motivation, with each item scored on a 5-point Likert scale ranging from 0 (not true for me) to 4 (very true for me). The Behavioral Regulations in Exercise Questionnaire v.3 has strong internal consistency for each motivational subscale; it validly measures the different types of motivation and is predictive of exercise behavior [68,69]. Eligible participants receive a Fitbit to wear for 1 week to assess baseline PA levels.

### Study Visit 2

At the start of this remote or in person visit, study staff evaluate the Fitbit wear compliance. Participants who did not wear the Fitbit for at least 3 weekdays and 1 weekend day are required to rewear the device. Participants who self-report <60 minutes of moderate to vigorous PA on the International Physical Activity Questionnaire are eligible. Eligible participants are randomized to one of the 2 conditions, the MobileMen intervention group or the Stridekick comparator group, as directed by the next assignment letter contained in a randomized sequence of sealed and numbered envelopes. Using a computerized pseudorandom number generator, the statistician determines the randomization order in advance. The study coordinator reveals the randomization after the participant is deemed eligible. Project staff assist each participant in downloading the designated (MobileMen or Stridekick) app onto their smartphone, provide an app orientation, and assist in app navigation.

### Study Visit 3

At the conclusion of the 6-month intervention, participants complete all follow-up assessments (except for the Physical Activity Readiness Questionnaire for Everyone and demographic questionnaires) by using the same protocols as in study visit 1. Participants in both groups also complete additional measures such as a survey to assess their satisfaction with the mobile app and the System Usability Scale, a validated 10-item measure that provides a global view of the subjective usability for technology, to assess the app’s usability [70]. Participants also complete a brief interview with the study staff to collect feedback regarding suggestions for app improvement and users’ impressions of the app content.

### Outcome Measures

#### Primary Outcomes

The primary outcome measure for this study is change in step counts derived from the Fitbit collected before the intervention (week 0) and after the intervention (week 24).

#### Secondary Outcomes

Time spent in moderate to vigorous PA, also derived from the Fitbit preintervention and postintervention data, is a secondary outcome measure. Other secondary outcomes include changes in body weight, blood pressure, quality of life, and healthy eating index score. Daily PA engagement assessed with Fitbit Charge 6 is another secondary outcome measure. Using the Fitbit application programming interface, study staff will access participant daily steps, time spent engaging in PA, number of PA bouts, and minutes spent in each Fitbit-defined heart rate zone. Although Fitbit’s thresholds and algorithms are proprietary, research indicates that minutes categorized in Fitbit data as “active” (fairly active plus very active) generally correspond to what we consider moderate to vigorous PA [71-73]. Usability (using the System Usability Scale) and overall satisfaction with the MobileMen app are the additional secondary outcomes.

#### Potential Mediators

We will measure autonomous motivation and self-efficacy for PA preintervention and postintervention as potential mediators [66-70].

#### Potential Covariates

Demographic variables, including age, marital status, education, family income, occupation, and employment status, will be tested as potential covariates in our statistical models. We will also examine BMI and the self-reported measured blood pressure (mm Hg) at baseline as covariates.

### Sample Size and Power Calculation

A sample of 50 participants per trial arm was determined based on step count data from a recent meta-analysis [72]. The standard deviation in nondiseased populations on their step counts is 2000 to 3000 steps per day. When the sample size per group is 50, a 2-sided 95% CI for the difference of two means will extend to about 800 steps per day from the observed difference in means, assuming an SD of 2000 steps per day and the CI is based on the large sample *z* statistic. Doubling the sample reduces the half width of the CI to 560 steps per day, which reduces the half width by only 30% but disproportionately increases the recruitment time and difficulty. Within the 2 arms, we can estimate longer term adherence at 6 months with a width of a 2-sided 95% CI equal to 578 steps per day when the sample proportion adherent is 0.50, and the width shortens as the adherence increases.

### Statistical Analyses

Response frequency, proportions, and means will be calculated. Means and standard deviations, medians and interquartile ranges, distributions, frequencies, and percentages will characterize the data. Comparisons on demographics (ie, age, marital status) will be done using chi-square tests, *t* tests, and correlations, with 2-tailed *P*=.05. The changes from baseline to all postbaseline visits until week 24 in steps per day will be assessed. The model includes response data from all postbaseline visits with no imputation for missing data, as the model assumes missingness at random. The step counts at baseline are included as a fixed factor in the model. An unstructured covariance structure is assumed, and the denominator degrees of freedom is computed using the Kenward-Roger method. In case the model will not converge with the unstructured covariance structure, the heterogeneous compound symmetry and the heterogeneous Toeplitz structure is used instead (in that order). The least-squares mean estimates for the mean change from baseline to week 24 as well as the difference in the estimates at 12 weeks is displayed with their corresponding standard errors, *P* values, and 95% CI. The characteristics of the dropouts and/or noncompliant individuals are intensively examined to shed light on the potential weaknesses of the MobileMen app (differential dropout overall or within certain subgroups) and assumptions about missingness in the estimates. The evaluation of the app will include its impact using the pre-post data. The examination of the correlation matrices along with the mean changes will inform whether the variables appear to move similarly in both arms and how the interventions are working.

To identify possible mediating constructs, we plan to assess preintervention and postintervention SDT motivation and self-efficacy for PA. The selected mediation variables will be assessed using PROC CAUSALMED, which provides estimates of direct, indirect, and total effects. We will be able to judge if mediation is occurring, and if so, the strength and direction of the mediation effect. Since this study may not have an adequate sample size to power mediation effectively, this analysis will be primarily used to estimate the potential effect in a larger sample for future trials.

App utilization data will be examined to understand the acceptability and intensity of the interactions with MobileMen via several criteria, including but not limited to logins, notifications sent and received, number of activities inputted, badges earned, tips read, number of health parameters inputted, competitions competed in, and incentives earned. Complier average causal effect models will be used to examine the intensity of the intervention app utilization [73].

## Results

The development of the fully functional MobileMen app began in September 2022, and the app was completed in July 2024. The design and implementation of CET are led by the project principal investigator (RLNJ). Rolling recruitment and enrollment of participants began in October 2024 and is planned to be completed by June 2025. The trial will be completed in the first half of 2025, with analyses completed in September 2025.

## Discussion

### Potential Findings

There are no PA promotion apps designed specifically for African American men. The MobileMen app seeks to fill this gap by becoming the first smartphone app that aims to support PA adoption and maintenance in African American men. We anticipate that app usage will be high in African American men given that it contains content that was requested, including the ability to track activity, nutrition information, goal setting, competitions, communication with other men, and rewards. We also anticipate that MobileMen will result in increased PA compared to men utilizing the Stridekick app. Finally, it is expected that the men utilizing the MobileMen app will show increases in the theoretical components targeted, which may serve as mediators of effects. If these findings are shown, it will provide evidence that a tailored app can be effective in promoting health behavior change in African American men. The next step would be to find ways of disseminating and implementing the app broadly through health systems or community-based organizations during which it will have the potential to improve the health of African American men nationwide.

### Comparison to Prior Work

To the best of our knowledge, MobileMen is a first of its kind intervention to promote PA in African American men. Existing literature demonstrates the importance of culturally tailored health interventions, particularly for populations that face unique structural and social barriers to health. Culturally tailored interventions have been shown to improve engagement, relevance, and outcomes in public health settings [56,57]. Despite the disproportionate burden of chronic disease among African American men, few health behavior change programs are specifically designed for this group [74]. African American men also face distinct barriers to PA related to gender role expectations and environmental constraints, making generalized PA programs less effective for this population [21]. The MobileMen app addresses this gap by providing a tailored solution grounded in the health behavior change theory and designed specifically for African American men. Additionally, African American people show high levels of engagement with mobile technology, supporting the use of mHealth tools as an accessible and culturally relevant strategy for promoting behavior change [75]. This focus on a high-engagement demographic with unique health needs sets MobileMen apart from existing health apps and underscores its potential value in fostering sustainable PA behavior change.

### Strengths and Limitations

The CET, which is currently in progress, has several strengths, including a multidisciplinary research team with combined expertise in African American men’s health, mHealth app development, PA assessment, wearable technology use in interventions, and psychological mediators of PA behavior change. The app was designed using a community-engaged approach [40]. The primary outcome of CET, that is, change in daily step count, will be assessed using objective activity monitoring rather than self-report, which is a study strength due to the reliability and validity of objectively measured step counts [76]. Further, this study not only aims to assess the primary PA outcome (steps) but also the hypothesized mediating construct (autonomous motivation), addressing the question of how MobileMen might support PA behavior change.

However, no study is without limitations. Even with a sample of 100 participants, we are underpowered to conduct statistical mediation tests, leaving us unable to assert if behavioral changes associated with MobileMen use can be attributed to changes in potential mediators. At present, the mechanisms of action for PA behavior change or most health behavior changes are not well understood, as they have not been closely examined [77]. Therefore, this is a critical next step for PA research in this population. Further, the location from which African American men are recruited, the Southern United States, presents a limitation in the generalizability of the results of the study. As such, a larger study with a more geographically diverse sample of African American men should be considered. Further, the mobile app is only available to men who have use of a mobile phone; nonmobile phone users will need other methods for accessing PA-tailored interventions. Finally, choosing a commercially available comparator app may introduce bias. Stridekick was not created by our team, and although we vetted the app, it is possible that components that were unaccounted for may influence our results. Further, differences in the content, degree of personalization, and theoretical foundation between Stridekick and MobileMen may introduce potential confounding. Therefore, any observed effects may partially reflect these differences rather than the cultural tailoring or theoretical elements of MobileMen alone. Further, the participants are aware of which app they are assigned to, which may introduce expectation bias. In summary, future studies of MobileMen might consider a comparator app that is more closely matched in content and theoretical underpinnings while also considering blinding or other strategies to reduce expectation bias.

### Conclusions

To date, no mobile PA and health app exists that is specifically tailored to the needs of African American men. MobileMen seeks to fill this gap with culturally tailored content and foundations in the health behavior change theory. This CET will assess its efficacy compared to a popular mHealth app, providing the research team with important data for app modifications before testing the final version in a large-scale effectiveness trial.

## Appendix

Multimedia Appendix 1 SPRINT 2025 Checklist.

## Abbreviations

CET: comparative effectiveness trial
mHealth: mobile health
PA: physical activity
SCT: social cognitive theory
SDT: self-determination theory

## References

[1] Thorpe RJ, Wilson-Frederick SM, Bowie JV, Coa K, Clay OJ, LaVeist TA, Whitfield KE (2013). Health behaviors and all-cause mortality in African American men. Am J Mens Health.

[2] Ogden CL, Carroll MD, Flegal KM (2014). Prevalence of obesity in the United States. JAMA.

[3] Cowie Catherine C, Rust Keith F, Byrd-Holt Danita D, Gregg Edward W, Ford Earl S, Geiss Linda S, Bainbridge Kathleen E, Fradkin Judith E (2010). Prevalence of diabetes and high risk for diabetes using A1C criteria in the U.S. population in 1988-2006. Diabetes Care.

[4] Go Alan S, Mozaffarian Dariush, Roger Véronique L, Benjamin Emelia J, Berry Jarett D, Blaha Michael J, Dai Shifan, Ford Earl S, Fox Caroline S, Franco Sheila, Fullerton Heather J, Gillespie Cathleen, Hailpern Susan M, Heit John A, Howard Virginia J, Huffman Mark D, Judd Suzanne E, Kissela Brett M, Kittner Steven J, Lackland Daniel T, Lichtman Judith H, Lisabeth Lynda D, Mackey Rachel H, Magid David J, Marcus Gregory M, Marelli Ariane, Matchar David B, McGuire Darren K, Mohler Emile R, Moy Claudia S, Mussolino Michael E, Neumar Robert W, Nichol Graham, Pandey Dilip K, Paynter Nina P, Reeves Matthew J, Sorlie Paul D, Stein Joel, Towfighi Amytis, Turan Tanya N, Virani Salim S, Wong Nathan D, Woo Daniel, Turner Melanie B, American Heart Association Statistics CommitteeStroke Statistics Subcommittee (2014). Heart disease and stroke statistics--2014 update: a report from the American Heart Association. Circulation.

[5] Sieverdes JC, Sui X, Lee D, Church TS, McClain A, Hand GA, Blair SN (2010). Physical activity, cardiorespiratory fitness and the incidence of type 2 diabetes in a prospective study of men. Br J Sports Med.

[6] Sui Xuemei, Hooker Steven P, Lee I-Min, Church Timothy S, Colabianchi Natalie, Lee Chong-Do, Blair Steven N (2008). A prospective study of cardiorespiratory fitness and risk of type 2 diabetes in women. Diabetes Care.

[7] Lee I, Djoussé Luc, Sesso Howard D, Wang Lu, Buring Julie E (2010). Physical activity and weight gain prevention. JAMA.

[8] Seo D, Li K (2010). Leisure-time physical activity dose-response effects on obesity among US adults: results from the 1999-2006 National Health and Nutrition Examination Survey. J Epidemiol Community Health.

[9] Lee I-Min, Wolin Kathleen Y, Freeman Sarah E, Sattlemair Jacob, Sesso Howard D (2014). Physical activity and survival after cancer diagnosis in men. J Phys Act Health.

[10] Hayes C, Kriska A (2008). Role of physical activity in diabetes management and prevention. J Am Diet Assoc.

[11] Williams Paul T (2008). Vigorous exercise, fitness and incident hypertension, high cholesterol, and diabetes. Med Sci Sports Exerc.

[12] Swift DL, Staiano AE, Johannsen NM, Lavie CJ, Earnest CP, Katzmarzyk PT, Blair SN, Newton RL, Church TS (2013). Low cardiorespiratory fitness in African Americans: a health disparity risk factor?. Sports Med.

[13] Hooker SP, Hutto B, Zhu W, Blair SN, Colabianchi N, Vena JE, Rhodes D, Howard VJ (2016). Accelerometer measured sedentary behavior and physical activity in white and black adults: The REGARDS study. J Sci Med Sport.

[14] Piercy Katrina L, Troiano Richard P, Ballard Rachel M, Carlson Susan A, Fulton Janet E, Galuska Deborah A, George Stephanie M, Olson Richard D (2018). The Physical Activity Guidelines for Americans. JAMA.

[15] Troiano Richard P, Berrigan David, Dodd Kevin W, Mâsse Louise C, Tilert Timothy, McDowell Margaret (2008). Physical activity in the United States measured by accelerometer. Med Sci Sports Exerc.

[16] Patel NA, Kianoush S, Jia X, Nambi V, Koh S, Patel J, Saeed A, Ahmed A, Al-Mallah M, Agarwala A, Virani S, Al Rifai M (2022). Racial/ethnic disparities and determinants of sufficient physical activity levels. Kans J Med.

[17] Macera Caroline A, Ham Sandra A, Yore Michelle M, Jones Deborah A, Ainsworth Barbara E, Kimsey C Dexter, Kohl Harold W (2005). Prevalence of physical activity in the United States: Behavioral Risk Factor Surveillance System, 2001. Prev Chronic Dis.

[18] Whitt-Glover MC, Taylor WC, Heath GW, Macera CA (2007). Self-reported physical activity among blacks: estimates from national surveys. Am J Prev Med.

[19] Powell LM, Slater S, Chaloupka FJ, Harper D (2006). Availability of physical activity-related facilities and neighborhood demographic and socioeconomic characteristics: a national study. Am J Public Health.

[20] Griffith DM, Gunter K, Watkins DC (2012). Measuring masculinity in research on men of color: findings and future directions. Am J Public Health.

[21] Griffith DM, Gunter K, Allen JO (2011). Male gender role strain as a barrier to African American men's physical activity. Health Educ Behav.

[22] Hammond WP, Matthews D, Mohottige D, Agyemang A, Corbie-Smith G (2010). Masculinity, medical mistrust, and preventive health services delays among community-dwelling African-American men. J Gen Intern Med.

[23] Corbie-Smith G, Thomas SB, St George Diane Marie M (2002). Distrust, race, and research. Arch Intern Med.

[24] (2011). mHealth: new horizons for health through mobile technologies. World Health Organization.

[25] Racial and ethnic differences in how people use mobile technology. Pew Research Center.

[26] Demographics of mobile device ownership and adoption in the United States. Pew Research Center.

[27] Guerriero C, Cairns J, Roberts I, Rodgers A, Whittaker R, Free C (2013). The cost-effectiveness of smoking cessation support delivered by mobile phone text messaging: Txt2stop. Eur J Health Econ.

[28] Burner ER, Menchine MD, Kubicek K, Robles M, Arora S (2014). Perceptions of successful cues to action and opportunities to augment behavioral triggers in diabetes self-management: qualitative analysis of a mobile intervention for low-income Latinos with diabetes. J Med Internet Res.

[29] Sharifi M, Dryden EM, Horan CM, Price S, Marshall R, Hacker K, Finkelstein JA, Taveras EM (2013). Leveraging text messaging and mobile technology to support pediatric obesity-related behavior change: a qualitative study using parent focus groups and interviews. J Med Internet Res.

[30] Tate EB, Spruijt-Metz D, O’Reilly G, Jordan-Marsh M, Gotsis M, Pentz MA, Dunton GF (2013). mHealth approaches to child obesity prevention: successes, unique challenges, and next directions. Behav Med Pract Policy Res.

[31] Cole-Lewis H, Kershaw T (2010). Text messaging as a tool for behavior change in disease prevention and management. Epidemiol Rev.

[32] James DC, Harville C, Sears C, Efunbumi O, Bondoc I (2017). Participation of African Americans in eHealth and mHealth studies: a systematic review. Telemed J E Health.

[33] Newton Robert L, Marker AM, Allen HR, Machtmes R, Han H, Johnson WD, Schuna John M, Broyles ST, Tudor-Locke C, Church TS (2014). Parent-targeted mobile phone intervention to increase physical activity in sedentary children: randomized pilot trial. JMIR Mhealth Uhealth.

[34] Schoeppe S, Alley S, Van Lippevelde W, Bray NA, Williams SL, Duncan MJ, Vandelanotte C (2016). Efficacy of interventions that use apps to improve diet, physical activity and sedentary behaviour: a systematic review. Int J Behav Nutr Phys Act.

[35] Riffenburg Kaitlyn M, Spartano Nicole L (2018). Physical activity and weight maintenance: the utility of wearable devices and mobile health technology in research and clinical settings. Curr Opin Endocrinol Diabetes Obes.

[36] Haberlin C, O’Dwyer T, Mockler D, Moran J, O’Donnell DM, Broderick J (2018). The use of eHealth to promote physical activity in cancer survivors: a systematic review. Support Care Cancer.

[37] Cyriac J, Jenkins S, Patten CA, Hayes SN, Jones C, Cooper LA, Brewer LC (2021). Improvements in diet and physical activity? Related psychosocial factors among African Americans using a mobile health lifestyle intervention to promote cardiovascular health: The FAITH!(fostering African American improvement in total health) app pilot study. JMIR Mhealth Uhealth.

[38] McCoy P, Leggett S, Bhuiyan A, Brown D, Frye P, Williams B (2017). Text messaging: an intervention to increase physical activity among African American participants in a faith-based, competitive weight loss program. Int J Environ Res Public Health.

[39] Nundy S, Razi RR, Dick JJ, Smith B, Mayo A, O'Connor A, Meltzer DO (2013). A text messaging intervention to improve heart failure self-management after hospital discharge in a largely African-American population: before-after study. J Med Internet Res.

[40] Brice Amanda, Fullmer Steve, Barger Charles, Serbinski Joel, Gallik Michael, Nauta Phillip, Swift Damon L, Stull April J, Buller David B, Griffith Derek M, Nuss Kayla, Newton Jr. Robert L (2025). MobileMen: The development of a mobile application to promote physical activity in African American men. Mhealth (forthcoming).

[41] Bandura A, Freeman WH, Lightsey R (1999). Self-efficacy: the exercise of control. J Cogn Psychother.

[42] Spencer MS, Rosland A, Kieffer EC, Sinco BR, Valerio M, Palmisano G, Anderson M, Guzman JR, Heisler M (2011). Effectiveness of a community health worker intervention among African American and Latino adults with type 2 diabetes: a randomized controlled trial. Am J Public Health.

[43] Ogedegbe G, Chaplin W, Schoenthaler A, Statman D, Berger D, Richardson T, Phillips E, Spencer J, Allegrante JP (2008). A practice-based trial of motivational interviewing and adherence in hypertensive African Americans. Am J Hypertens.

[44] Resnicow K, Jackson A, Wang T, De AK, McCarty F, Dudley WN, Baranowski T (2001). A motivational interviewing intervention to increase fruit and vegetable intake through Black churches: results of the Eat for Life trial. Am J Public Health.

[45] Kumanyika Shiriki K, Fassbender Jennifer E, Sarwer David B, Phipps Etienne, Allison Kelly C, Localio Russell, Morales Knashawn H, Wesby Lisa, Harralson Tina, Kessler Ronni, Tan-Torres Susan, Han Xiaoyan, Tsai Adam G, Wadden Thomas A (2012). One-year results of the Think Health! study of weight management in primary care practices. Obesity (Silver Spring).

[46] Boltri JM, Davis-Smith M, Okosun IS, Seale J Paul, Foster B (2011). Translation of the national institutes of health diabetes prevention program in African American churches. J Natl Med Assoc.

[47] Newton Robert L, Perri Michael G (2004). A randomized pilot trial of exercise promotion in sedentary African-American adults. Ethn Dis.

[48] Markland D, Ryan RM, Tobin VJ, Rollnick S (2005). Motivational interviewing and self-determination theory. J Soc Clin Psychol.

[49] Silva MN, Markland D, Minderico CS, Vieira PN, Castro MM, Coutinho SR, Santos TC, Matos MG, Sardinha LB, Teixeira PJ (2008). A randomized controlled trial to evaluate self-determination theory for exercise adherence and weight control: rationale and intervention description. BMC Public Health.

[50] Silva MN, Vieira PN, Coutinho SR, Minderico CS, Matos MG, Sardinha LB, Teixeira PJ (2010). Using self-determination theory to promote physical activity and weight control: a randomized controlled trial in women. J Behav Med.

[51] Ryan Richard M, Patrick Heather, Deci Edward L, Williams Geoffrey C (2008). Facilitating health behaviour change and its maintenance: interventions based on self-determination theory. The European Health Psychologist.

[52] Vansteenkiste M, Soenens B, Vandereycken W (2005). Motivation to change in eating disorder patients: a conceptual clarification on the basis of self-determination theory. Int J Eat Disord.

[53] Teixeira PJ, Carraça Eliana V, Markland D, Silva MN, Ryan RM (2012). Exercise, physical activity, and self-determination theory: a systematic review. Int J Behav Nutr Phys Act.

[54] Griffith Derek M, Jaeger Emily C (2020). Mighty men: A faith-based weight loss intervention to reduce cancer risk in African American men. Adv Cancer Res.

[55] Villalobos-Zúñiga G, Cherubini M (2020). Apps that motivate: a taxonomy of app features based on self-determination theory. Int J Hum Comput Stud.

[56] Kreuter MW, Lukwago SN, Bucholtz DC, Clark EM, Sanders-Thompson V (2003). Achieving cultural appropriateness in health promotion programs: targeted and tailored approaches. Health Educ Behav.

[57] Resnicow K, Baranowski T, Ahluwalia J S, Braithwaite R L (1999). Cultural sensitivity in public health: defined and demystified. Ethn Dis.

[58] Hooker SP, Harmon B, Burroughs EL, Rheaume CE, Wilcox S (2011). Exploring the feasibility of a physical activity intervention for midlife African American men. Health Educ Res.

[59] Porche DJ (2012). Men: the missing client in family planning. Am J Mens Health.

[60] Newton Robert L, Carter L, St Romain Jessica, Jerrod T, Griffith DM, Myers V (2019). Development of a mobile phone app to maintain physical activity in African American men. Mhealth.

[61] Tudor-Locke C, Bassett DR (2004). How many steps/day are enough? Preliminary pedometer indices for public health. Sports Med.

[62] Ware JE, Sherbourne CD (1992). The MOS 36-ltem Short-Form Health Survey (SF-36). Medical Care.

[63] Wolinsky FD, Miller DK, Andresen EM, Malmstrom TK, Miller JP (2004). Health-related quality of life in middle-aged African Americans. J Gerontol B Psychol Sci Soc Sci.

[64] Craig CL, Marshall AL, Sjöström Michael, Bauman AE, Booth ML, Ainsworth BE, Pratt M, Ekelund U, Yngve A, Sallis JF, Oja P (2003). International physical activity questionnaire: 12-country reliability and validity. Med Sci Sports Exerc.

[65] Shams-White Marissa M, Pannucci TusaRebecca E, Lerman Jennifer L, Herrick Kirsten A, Zimmer Meghan, Meyers Mathieu Kevin, Stoody Eve E, Reedy Jill (2023). Healthy Eating Index-2020: review and update process to reflect the dietary guidelines for Americans, 2020-2025. J Acad Nutr Diet.

[66] Henry H, Reimer K, Smith C, Reicks M (2006). Associations of decisional balance, processes of change, and self-efficacy with stages of change for increased fruit and vegetable intake among low-income, African-American mothers. J Am Diet Assoc.

[67] Sallis JF, Pinski RB, Grossman RM, Patterson TL, Nader PR (1988). The development of self-efficacy scales for healthrelated diet and exercise behaviors. Health Educ Res.

[68] Markland David, Tobin Vannessa (2004). A modification to the behavioural regulation in exercise questionnaire to include an assessment of amotivation. J Sport Exerc Psychol.

[69] Wilson PM, Rodgers WM, Loitz CC, Scime G (2007). “It's Who I Am … Really!’ The importance of integrated regulation in exercise contexts 1. J Appl Biobehavioral Res.

[70] Brooke J (1996). SUS: A quick and dirty usability scale. Usability Evaluation in Industry.

[71] Hartman SJ, Chen R, Tam RM, Narayan HK, Natarajan L, Liu L (2022). Fitbit use and activity levels from intervention to 2 years after: secondary analysis of a randomized controlled trial. JMIR Mhealth Uhealth.

[72] Wang C, Lizardo O, Hachen DS (2021). Using Fitbit data to examine factors that affect daily activity levels of college students. PLoS One.

[73] Semanik Pamela, Lee Jungwha, Pellegrini Christine A, Song Jing, Dunlop Dorothy D, Chang Rowland W (2020). Comparison of physical activity measures derived from the Fitbit Flex and the ActiGraph GT3X+ in an employee population with chronic knee symptoms. ACR Open Rheumatol.

[74] Whitt-Glover M C, Keith NR, Ceaser TG, Virgil K, Ledford L, Hasson RE (2014). A systematic review of physical activity interventions among African American adults: evidence from 2009 to 2013. Obes Rev.

[75] Joseph RP, Ainsworth BE, Keller C, Dodgson JE (2015). Barriers to physical activity among African American women: an integrative review of the literature. Women Health.

[76] Fiedler J, Eckert T, Burchartz A, Woll A, Wunsch K (2021). Comparison of self-reported and device-based measured physical activity using measures of stability, reliability, and validity in adults and children. Sensors (Basel).

[77] Nielsen L, Riddle M, King JW, Aklin Will M, Chen Wen, Clark David, Collier Elaine, Czajkowski Susan, Esposito Layla, Ferrer Rebecca, Green Paige, Hunter Christine, Kehl Karen, King Rosalind, Onken Lisa, Simmons Janine M, Stoeckel Luke, Stoney Catherine, Tully Lois, Weber Wendy, NIH Science of Behavior Change Implementation Team (2018). The NIH Science of Behavior Change Program: transforming the science through a focus on mechanisms of change. Behav Res Ther.

